# Evaluation of Human Milk Microbiota by 16S rRNA Gene Next-Generation Sequencing (NGS) and Cultivation/MALDI-TOF Mass Spectrometry Identification

**DOI:** 10.3389/fmicb.2019.02612

**Published:** 2019-11-15

**Authors:** Primož Treven, Aleksander Mahnič, Maja Rupnik, Majda Golob, Tina Pirš, Bojana Bogovič Matijašić, Petra Mohar Lorbeg

**Affiliations:** ^1^Department of Animal Science, Biotechnical Faculty, Institute of Dairy Science and Probiotics, University of Ljubljana, Ljubljana, Slovenia; ^2^National Laboratory of Health, Environment and Food, Maribor, Slovenia; ^3^Faculty of Medicine, University of Maribor, Maribor, Slovenia; ^4^Faculty of Veterinary Medicine, Institute of Microbiology and Parasitology, University of Ljubljana, Ljubljana, Slovenia

**Keywords:** milk microbiota, microbiota assembly, breastfeeding, breast pump, probiotic drops

## Abstract

The aim of the present study was to characterize human milk microbiota (HMM) with 16S rRNA gene amplicon next-generation sequencing and cultivation/matrix-assisted laser desorption/ionization (MALDI)-time of flight (TOF) mass spectrometry (MS) identification approaches. We analyzed 31 human milk samples from healthy Slovenian mothers. To check the accuracy of MALDI-TOF MS identification, several colonies representing most abundant genera and those, which could not be reliably identified by MALDI-TOF, were subjected to Sanger sequencing of their 16S rRNA gene. We showed that cultivation/MALDI-TOF MS was a suitable tool for culture-dependent determination of HMM. With both approaches, *Staphylococcus* and *Streptococcus* were found as predominant genera in HMM and the abundance of *Staphylococcus* was associated with decreased microbial diversity. In addition, we characterized factors that might influence HMM. The use of a breast pump was significantly associated with composition of HMM, higher microbial load, and lower abundance of cultivable staphylococci. Moreover, our study suggests that administration of probiotics to the suckling infant might influence HMM by increased abundance of lactobacilli and the presence of viable probiotic bacteria in human milk. However, since our study was observational with relatively small sample size, more targeted studies are needed to study possible transfer of probiotics to the mammary gland via an external route and the physiological relevance of these events.

## Introduction

Majority of bacteria found in breastfed infants’ gut, including gut-associated obligate anaerobes, originate from human milk (Jost et al., 2013b; Pannaraj et al., 2017). Many studies using culture-dependent and -independent techniques have reported that breast milk harbors bacterial genera such as *Streptococcus*, *Staphylococcus*, propionibacteria, *Clostridium*, *Serratia*, *Weissella*, bifidobacteria, and lactic acid bacteria (Jeurink et al., 2013; Jost et al., 2013a; Fitzstevens et al., 2017).

Individual factors that shape the human milk microbiota (HMM) are still unclear since reports seem to be contradictory. Several recent studies using next-generation sequencing (NGS) and classical culture-based methods have pointed out geographical location (Cabrera-Rubio et al., 2016; Li et al., 2017; Xi et al., 2017), mode of delivery (Cabrera-Rubio et al., 2012, 2016; Khodayar-Pardo et al., 2014; Kumar et al., 2016; Li et al., 2017; Williams et al., 2017a; Xi et al., 2017), maternal lifestyle (Vaidya et al., 2017), lactation period (Cabrera-Rubio et al., 2012; Khodayar-Pardo et al., 2014; Xi et al., 2017; Chen et al., 2018; Simpson et al., 2018), maternal dietary intake or antibiotic therapy (Soto et al., 2014; Williams et al., 2017a, b), maternal body mass index (BMI) (Cabrera-Rubio et al., 2012; Kumar et al., 2016; Williams et al., 2017a), mode of breastfeeding (Moossavi et al., 2019), and mastitis (Jimenez et al., 2015; Patel et al., 2017) as factors that influence HMM. On the other side, some authors reported no differences in HMM regarding mode of delivery, geographic location (Pannaraj et al., 2017), maternal BMI (Li et al., 2017), and stage of lactation (Sakwinska et al., 2016; Li et al., 2017). Nevertheless, beside different study protocols in these reports, the human milk collection protocol seems to also be an important factor that could contribute to inconsistencies (Sakwinska et al., 2016).

Matrix-assisted laser desorption/ionization (MALDI)-time of flight (TOF) mass spectrometry (MS) is a rapid, sensitive, and automated system for bacterial identification. Generated protein profiles of the tested whole bacterial cells are compared with protein profiles of reference bacteria which are available in spectral database. More than 200 *Lactobacillus* spectra are already available in the MALDI Biotyper (Bruker Daltonics, Bremen, Germany) database and therefore is frequently used in the analysis of lactic acid bacteria from milk and dairy products (Albesharat et al., 2011; Angelakis et al., 2011; Dušková et al., 2012; Delavenne et al., 2013; Bunesova et al., 2014; Nacef et al., 2016). Moreover, continuously increasing and frequently updated commercial databases improve the identification of different micro-organisms found in dairy industry (Čanžek Majhenič et al., 2017). MALDI-TOF MS profiling has become a favored tool for the analysis of environmental strains and microbial communities due to high-throughput capabilities, simple sample preparation, and low analysis costs (Kostrzewa et al., 2016).

The aim of the present study was to characterize HMM with culture-independent 16S rRNA gene NGS (16S NGS) and cultivation/MALDI-TOF MS techniques. In addition, we aimed to evaluate the accuracy of MALDI-TOF MS identification technique for determination of HMM and to evaluate some of the factors that might influence HMM.

## Materials and Methods

### Subjects and Sample Collection

A total of 32 healthy mothers from the central Slovenian region donated one milk sample (at least 25 mL), between the 3rd and 8th week of lactation. This study was conducted according to the guidelines of the Declaration of Helsinki. All of the procedures involving human subjects were approved by the National Medical Ethics Committee of the Republic of Slovenia (0120-328/2017/3). Written informed consent was obtained from all participants who provided the milk samples. We entered and analyzed all samples and questionnaire data anonymously and published all data anonymously by using personal numbers. We excluded volunteers with autoimmune chronic illnesses, acute and chronic infections, and volunteers with pre-term delivery (≤37 weeks of pregnancy, with abnormal birth weight). Participating mothers were not treated with antibiotics at least 1 month before sampling, except one who completed antibiotic therapy 2 weeks before sample collection. After signing informed consent, mothers were asked to take the sample of milk from both breasts, by manual expression or with a breast pump (not provided). They were instructed to clean each breast with warm water and soap, discard the first drop before collecting the sample, and freeze the sample immediately after collection. At the same time, mothers were asked to complete a short questionnaire (Supplementary Tables S1, S12). Samples were transported to the laboratory within a week after collection and frozen at −70°C. One sample was excluded from the study due to low bacterial DNA and insufficient number of reads after 16S NGS.

### DNA Extraction From Human Milk Samples

Four milliliters of milk was spun down at 16,000 × *g* for 5 min at 4°C. Supernatant and the top fat layer were discarded and the remaining fat was removed from the microcentrifuge tube with sterile inoculation loop. The pellet was resuspended in 500 μL TE buffer (10 mM Tris–HCl, 1 mM EDTA, pH 8) containing 25 mg/mL lysozyme and 25 U/mL mutanolysin. The mixture was incubated at 37°C for 2 h. DNA extraction was performed with Maxwell^TM^ 16 Tissue DNA Purification kit (with Maxwell^TM^ 16 instrument; Promega, United States). For experimental controls, instead of milk sample, we used 4 mL of ultrapure water.

### Bacterial Count Estimates by Quantitative Real-Time PCR

Bacterial cell numbers were estimated using quantitative PCR (qPCR) as described previously with modifications (Obermajer et al., 2015). DNA for real-time PCR standard curve was isolated from overnight bacterial culture of *Staphylococcus epidermidis* IM 385, *S. epidermidis* were cultivated for 18 h in BHI medium at 37°C in aerobic conditions. Cell numbers were determined by plating serial dilutions of the exponential-phase cells onto BHI agar for colony enumeration. Twofold dilution series of DNA isolated directly from 18-h culture were prepared and amplified by real-time PCR. PCR reaction was performed in 20-μL volume containing 1× KAPA SYBR FAST Universal 2× qPCR Master Mix (Kapa Biosystems, United States), 0.2 μM of each specific primer, and 5 μL of extracted gDNA. Primers 331F (5′-TCCTACGGGAGGCAGCAGT-3′) and 797R (5′-GGACTACCAGGGTATCTAATCCTGTT-3′) were specific for V3–V4 variable region of the bacterial 16S rRNA coding genes and described previously (Nadkarni et al., 2002). PCR amplification was performed with an MX3000P instrument (Stratagene, United States). The amplification program was 50°C for 2 min and 95°C for 2 min, 30 cycles of 95°C for 30 s, 60°C for 30 s, and 72°C for 30 s. All reactions were subjected also to melting curve analysis in order to establish the specificity of the amplification. Correlation between Ct values and initial number of colony forming units was determined by Stratagene MX3000P system’s program (Stratagene, United States). Reaction efficiency (*E* = 92.2%) was calculated from the slope of the standard curve [*y* = −3.525 ^∗^ Log(*x*) + 36.26; *R*^2^ = 0.995] as *E* = 10^–1/slope^ − 1. Window of linearity was between 4.4 ^∗^ 10^3^ and 3.2 ^∗^ 10^6^ colony forming units equivalents. No signal above the detection threshold was obtained for the non-template control samples (Ct > 30).

### 16S rRNA Gene Next-Generation Sequencing

#### Library Preparation and Sequencing

16S sequencing was performed by targeting the V3–V4 hypervariable region of the 16S rRNA gene using broad range set of primers Bakt_341F (5′-CCTACGGGNGGCWGCAG-3′) – Bakt_805R (5′-GACTACHVGGGTATCTAATCC-3′) (Klindworth et al., 2013). Libraries were constructed according to recommended Illumina 16S Metagenomic Sequencing Library Preparation manual protocol (Illumina, CA, United States). Sequencing was performed on Illumina MiSeq platform with MiSeq Reagent Kit V3 (2 × 300 cycle, 10% PhiX).

#### Sequence Processing and Taxonomic Assignment

Quality filtering and taxonomic assignment was performed in mothur (v.1.36.1) (Schloss et al., 2009). The following criteria were implemented: (i) reads were not allowed any ambiguous bases and maximum homopolymer length was set to 8 base pairs; (ii) the reads were aligned against the Silva reference alignment (Release 123); (iii) chimeras were identified with the UCHIME algorithm; (iv) the taxonomy was assigned to reads according to the RDP training set (version 16) with 0.80 bootstrap threshold value; and (v) sequences were clustered into operational taxonomic units (OTUs) at the 97% similarity cut-off. After quality filtering we obtained an average depth of 53637 reads per sample (min 3106, max 117886). Unique reads which were represented in the abundance of <0.01% were removed. All samples were rarefied to 10,000 reads per sample. A single sample with <10,000 reads was removed from further analysis.

Analysis of alpha diversity (Chao1 index estimating community richness and Shannon index estimating community diversity), beta diversity (AMOVA, HOMOVA based on Bray–Curtis dissimilarity), and population level analysis [linear discriminant analysis (LDA) effect size (LEfSe) (Segata et al., 2011)] were performed in mothur (v.1.36.1). Oligotyping method was implemented as described in Eren et al. (2014) for a detailed analysis of reads in the instances, when the V3–V4 region was informative enough for the classification up to the species taxonomic level.

### Culture and Isolation

Samples were thawed, diluted 1:10 in buffered peptone water (Merck, Darmstadt, Germany), and plated on blood agar [BA; Columbia agar + 5% sheep blood (BioMérieux, Marcy l’Etoile, France)], tryptic soy agar (TSA; Biolife Italiana, Milano, Italy), Wilkins-Chalgrene anaerobe agar (WCA-M; Oxoid, Basingstoke, United Kingdom) supplemented with mupirocin (50 mg/L) (AppliChem GmbH, Germany) and Rogosa agar (ROG; pH 5.5; Merck, Darmstadt, Germany). Plates were incubated at 37°C for 72 h in aerobic (TSA) conditions or anaerobic conditions (BA, WCA-M and ROG). Colonies with various morphology were systematically picked up from TSA (15 colonies), BA (15 colonies), WCA, and ROG (up to 10 colonies) and their genus was determined by MALDI-TOF MS. Due to poor growth of bacteria on Rogosa agar, samples were re-analyzed by direct plating of 1 mL of milk sample.

### MALDI-TOF MS Identification

We used a direct-transfer method according to the manufacturer’s instructions and as previously described (Ledina et al., 2018). Prior to MALDI-TOF MS analyses, a small amount of bacterial colony from agar plates was picked and transferred onto a steel target plate and left to air-dry. Dried sample was covered with 1 μL of HCCA (a-cyano-4-hydroxycinnamic acid; Bruker Daltonics, Bremen, Germany). The ionization of the sample was performed in an automated mode with a laser beam that generated singly protonated ions from the analytes in the sample. We used recommended settings for bacterial species identification in a linear positive mode, at a laser frequency of 60 Hz within a mass range from 2 to 20 kDa (with highest resolution in mass range from 5 to 8 kDa); voltage values of ion source 1 was 20 kV and of ion source 2 18.2 kV; lens tension = 6 kV; pulsed ion extraction was set at a value 5000 V; and laser power was modulated between 10 and 40% (global attenuator offset: 12%; attenuator offset: 10%; attenuator range: 30%). Calibration of the mass spectrometer was performed with the Bruker’s bacterial test standard (*Escherichia coli* DH5a extracts with the additional proteins RNase A and myoglobin, Bruker Daltonics, Bremen, Germany). Automatic measurement of the spectrum and comparative analysis with reference spectra of bacteria were performed using the MALDI Biotyper 3.1 software (Bruker Daltonics, Bremen, Germany). The reliability of identification in the MALDI Biotyper system was expressed in points. Results obtained from the Microflex LT system were calculated as score values and associated-color code (green, yellow, and red). Score values exceeding 2.0 (green color) indicated a highly probable identification at the species level, while the score values between 1.7 and 2 (yellow color) were considered to be reliable at the genus level. In the case of ambiguous results generated for some isolates with score values <1.7 (red color), the extended direct-transfer method with 70% formic acid was employed.

### Verification of MALDI-TOF MS Determination With 16S rRNA and Sanger Sequencing

Several colonies representing most abundant genera and those marked as “no reliable identification (NRID)” were isolated and subjected to Sanger sequencing of their 16S rRNA. Selected colonies were cultivated in corresponding media and their DNA was extracted with Wizard Genomic DNA Purification Kit (Promega, United States) according to the manufacturer’s protocol for isolation of genomic DNA from Gram-positive bacteria. Complete 16S rRNA gene (primers 27f: 3′-AGAGTTTGATCCTGGCTCAG-5′ and 1495R: 3′-CTACGGCTACCTTGTTACGA-5′) was amplified as described by Yu et al. (2011) with modifications. DNA extracted from isolated bacteria was used (1 μL) as a template in a 25 μL reaction volume. Program of amplification constituted: 2 min of denaturation at 95°C followed by 30 cycles of denaturation (95°C, 1 min), annealing (59°C, 1 min), elongation (72°C, 2 min), and final elongation for 5 min at 72°C. Sanger sequencing of PCR products was performed by Microsynth AG (Vienna, Austria). Gained rRNA sequences were aligned and classified against the SILVA database and by using BLAST search.

### Detection of *Lacobacillus rhamnosus* GG an *Lactobacillus reuteri* DSM 17938 With Strain-Specific PCR

For detection of *Lactobacillus rhamnosus* GG (LGG) and *Lactobacillus reuteri* DSM 17938 (LRD) 1 mL of each milk sample was plated on ROG agar and incubated at 37°C for 72 h in anaerobic conditions. Each grown colony was isolated by cultivation in MRS broth. DNA from isolated bacteria was extracted with Wizard Genomic DNA Purification Kit (Promega, United States) according to the manufacturer’s protocol for isolation of genomic DNA from Gram-positive bacteria. For strain-specific PCR, DNA extracted from isolated bacteria was 100-fold diluted in water and used (1 μL) as a template in a 20-μL reaction volume. The LGG strain-specific PCR was performed as described previously (Ahlroos and Tynkkynen, 2009) by using end-point PCR with the forward primer 5′-CGCCCTTAACAGCAGTCTTCAAAT-3′ and reverse primer 5′-ACGCGCCCTCCGTATGCTTAAACC-3′. Strain-specific PCR primers 1694f (5′-TTAAGGATGCAAACCCGAAC-3′) and 1694r (5′-CCTTGTCACCTGGAACCACT-3′) were used to detect LRD (Rattanaprasert et al., 2014). Each reaction was initiated with 2 min of denaturation at 95°C followed by 30 cycles of denaturation (95°C), annealing (60 or 62°C for LGG and LRD, respectively), elongation (72°C) for 30 s, and finished with elongation for 5 min at 72°C. Amplification products were detected by electrophoresis in TAE buffer using a 1.2% agarose gel followed by staining in SYBR^®^ Safe DNA gel stain (Invitrogen, United States).

### Statistical Analysis

The statistics and graphic representation were done in R (version 3.1.3) using packages “ggplot2” and “vegan,” and SigmaPlot 11.0 (Systat Software; SPSS Inc., Chicago, IL, United States).

## Results

### Overview of the HMM – *Staphylococcus* and *Streptococcus* Are the Predominant Genera

**FIGURE 1:**
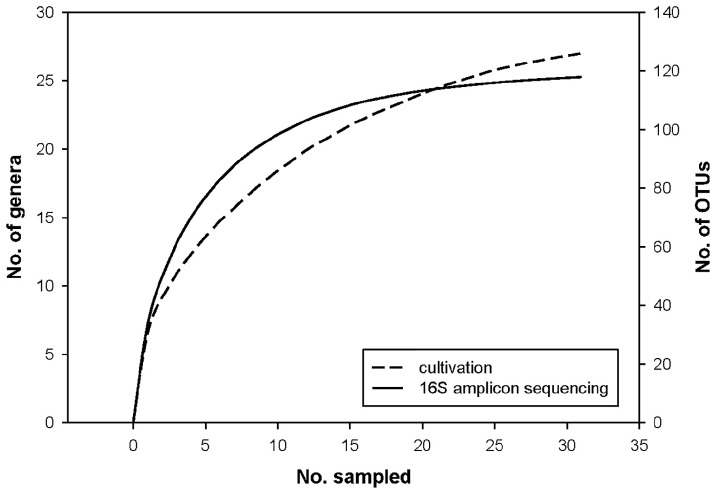
Rarefaction curves of the 16S metagenomics sequencing and the cultivation approaches.

We analyzed 31 human milk samples from self-reported healthy Slovenian mothers in the 3rd–8th week of lactation (Supplementary Table S1) with amplicon sequencing targeting V3–V4 variable region of the bacterial 16S rRNA gene and with cultivation/MALDI-TOF MS identification. After the quality filtering and rarefying all samples to 10,000 reads, we obtained 118 OTUs, assigned to 59 different genera (Supplementary Tables S2–S4). The mean number of observed OTUs was 33.26 (range 10–70) per sample with corresponding mean Chao1 richness index 38.37 (*SD* 12.66). With the cultivation approach, we analyzed 1086 colonies with an average of 35 (12–44) colonies per sample, resulting in determination of 25 different genera (Supplementary Table S5). The comparison of rarefaction curves of the 16S NGS and the cultivation approaches (Figure 1) suggested that on average 35 picked colonies per sample in 31 samples was too low, to capture the entire cultivable diversity of HMM. However, our systemic approach and usage of MALDI-TOF MS for identification enabled us to cover the major representatives of cultivable HMM.

**FIGURE 2:**
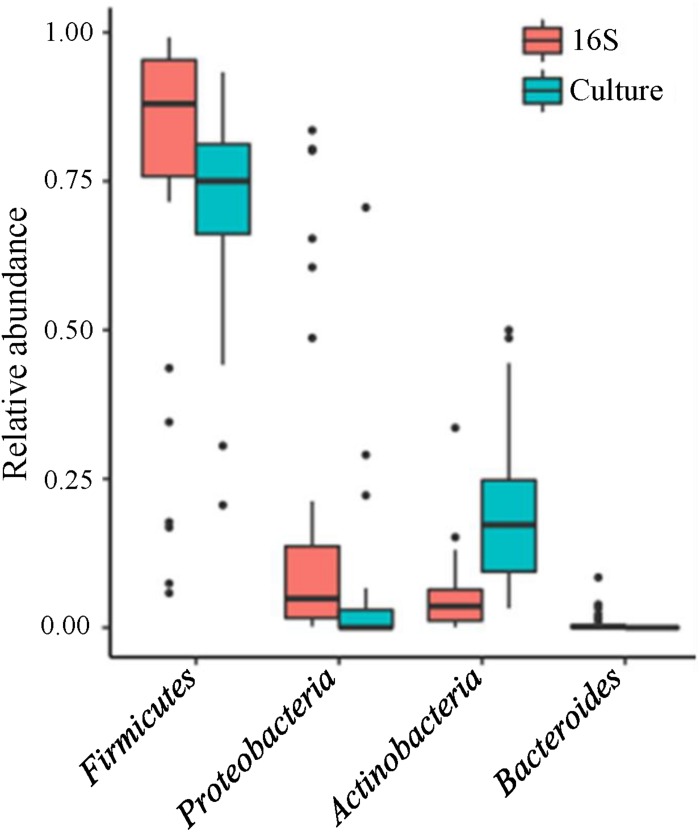
Relative abundance of bacterial phyla in human milk samples. Boxplot presentation of relative abundances of bacterial phyla shown for 16S rRNA gene NGS approach (red) and cultivation approach (blue). Only phyla present in relative abundance >0.1% are shown.

**FIGURE 3:**
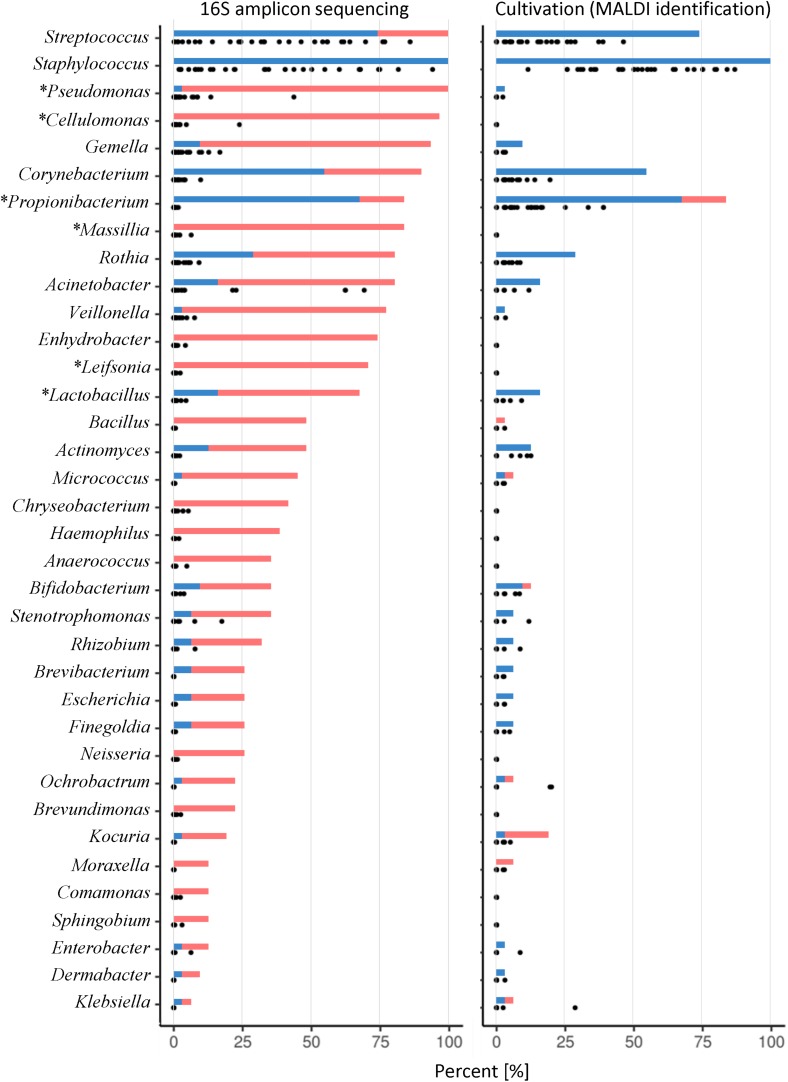
Prevalence and relative abundance of genera detected in HMM. Either 16S rRNA gene NGS **(left)** or cultivation with MALDI-TOF MS identification **(right)** were used. Graph presents all genera detected with 16S NGS with overall relative abundance >0.1% and additionally those detected with cultivation. Solid bars show the prevalence of a specific genus. The blue line shows the prevalence of samples where genus was detected with both sequencing and cultivation approach while the red line shows the prevalence of samples where genus was detected with only one approach [16S rRNA gene NGS **(left)** or cultivation **(right)**]. Dots represent the relative abundance for each analyzed sample separately (*n* = 31). An asterisk by the genus name indicates that at least one representative of genus was most likely present as a contamination.

The analyses of 16S data revealed *Firmicutes*, *Proteobacteria*, and *Actinobacteria* as the three most abundant phyla (Figure 2). At the genus level, 14 genera were detected in >50% of the samples (Figure 3, left panel). *Pseudomonas*, *Cellulomonas*, *Massilia*, and *Leifsonia* can be considered as contaminants since they were detected in high numbers in all experimental or library preparation controls. Their presence in analyzed milk samples is thus questionable, since their relative abundance in the majority of the samples was much lower compared to control samples (Supplementary Table S2). Moreover, by culture we detected only one colony of *Pseudomonas* in one sample, although these organisms are generally not fastidious and TSA agar is expected to support their growth. Disregarding *Pseudomonas*, only *Staphylococcus* and *Streptococcus* were detected in all samples, with average relative abundance of 36.0 and 35.6%, respectively. Other highly abundant genera, present in >50% of the samples, were *Acinetobacter* (8.3%, average relative abundance), *Gemella* (2.6%), *Rothia* (1.5%), *Corynebacterium* (1.3%), *Veillonella* (0.9%), *Lactobacillus* (0.4%), *Enhydrobacter* (0.3%), and *Propionibacterium* (0.2%). In the case of *Propionibacterium* and *Lactobacillus* one representative of genus was detected in experimental and library preparation controls, probably leading to slightly overestimated relative abundance.

The three most abundant phyla, determined with culture-based methods, were *Firmicutes*, *Actinobacteria*, and *Proteobacteria* (Figure 2). At the genus level, only *Staphylococcus* was detected in all samples (Figure 3, right panel) with the predominant average abundance (52.2%). Other more abundant genera, detected in >50% of the samples, were S*treptococcus* (12.8%), *Propionibacterium* (10.4%), and *Corynebacterium* (3.5%). Genera, present in >15% of the samples, were *Rothia*, *Kocuria*, *Acinetobacter*, *Lactobacillus*, *Actinomyces*, and *Bifidobacterium*.

**FIGURE 4:**
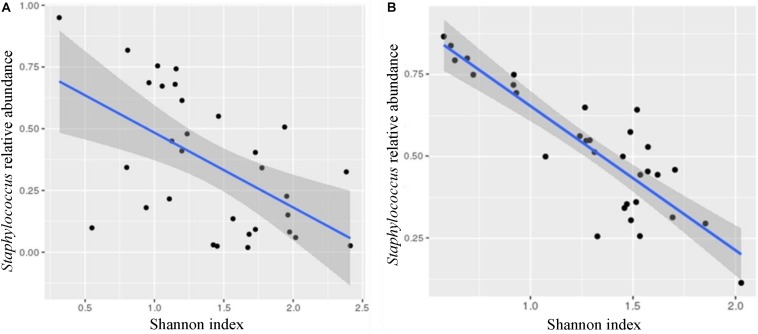
Correlations between *Staphylococcus* relative abundance and Shannon diversity index. The Shannon diversity index negatively correlated with the abundance of *Staphylococcus* using both methods, 16S metagenomic sequencing (**A**; Otu001; Pearson’s *r*: –0.556, *p* < 0.01^∗^) and cultivation (**B**; Pearson’s *r*: –0.629, *p* < 0.001). ^∗^*p*-value is not below the corrected *p*-value [Benjamini–Hochberg correction (FDR < 0.05) for multiple testing] and the correlation could not be considered as statistically significant.

Shannon diversity index showed negative correlation with the relative abundance of *Staphylococcus* (Otu001; Pearson’s *r*: −0.556, Figure 4A and Supplementary Table S6) and positive with the relative abundance of *Veillonella* (Otu014; Pearson’s *r*: 0.509). After implementing “Benjamini–Hochberg correction” (FDR < 0.05), these correlations did not prove to be statistically significant. However, when we used the cultivation approach, the correlation between Shannon diversity index and the abundance of *Staphylococcus* reached statistical significance (Pearson’s *r*: −0.629, *p* < 0.001; Figure 4B and Supplementary Table S7).

To check the accuracy of MALDI-TOF MS identification, several colonies representing most abundant genera and those marked as “NRID” were isolated and subjected to Sanger sequencing of their 16S rRNA gene (Supplementary Tables S8, S9). Except *Mycoplasma*, the genera of selected colonies matched with 16S rRNA Sanger sequencing results. Sanger sequencing of all 13 presumable *Mycoplasma* colonies showed that they belong to *Cutibacterium* (77%), *Rothia* (15%), and *Kocuria* (8%). All falsely detected *Mycoplasma* were picked from WCA-M agar plates. Tests of sole autoclaved WCA agar on MALDI-TOF MS also showed the presence of *Mycoplasma*, which suggests that this media could interfere with MALDI-TOF MS detection and generates similar spectra as *Mycoplasma* cells.

### Evaluation of Factors That Shape HMM

Different alpha and beta diversity analysis approaches were implemented to identify the influence of factors on HMM composition. Factors such as mother’s or infant’s health, use of medicines, geographical location, gestational age, and lactation stage were defined in inclusion criteria. From the analysis, we excluded type of delivery since only three mothers delivered with cesarean section (Supplementary Table S1). Factors such as mother’s age, maternal BMI, and baby’s gender were not significantly associated with the specific patterns in HMM, regardless of whether we used the cultivation approach or 16S NGS. Seven mothers reported the consumption of different types of probiotics such as probiotic supplements, yogurts, or cheeses 1 month before the sampling. LEfSe analysis showed significantly increased relative abundance of several OTUs (Table 1), all corresponding to the phylum of *Proteobacteria* (Otu004, Otu022, and Otu094) – however, the cultivation approach did not confirm these results.

**TABLE 1:** Association of factors with specific OTUs and genera detected in HMM with 16S NGS.

**Factor**	**Class**	**Number of samples**	**Genus (OTU)**	**LogMax mean**	**LDA**	***p*-value**
Milk collection technique	MANUAL	22	*Pseudomonas* (Otu006)	4.7136	4.3693	0.0003
			*Cellulomonas* (Otu008)	4.3273	3.9683	0.0002
			*Leifsonia* (Otu009)	3.4457	3.1532	0.0008
			*Massilia* (Otu010)	3.8316	3.5199	0.0007
			*Bacilaceae* (Otu021)	3.5045	3.1846	0.0002
			*Reylanella* (Otu032)	2.9498	2.8352	0.0020
			*Herbaspirillum* (Otu046)	2.8724	2.7494	0.0407
	PUMP	9	*Stenotrophomonas* (Otu017)	4.3792	4.0706	0.0093
			*Rhizobium* (Otu027)	4.0009	3.7721	0.0100
			*Actinomyces* (Otu040)	3.1918	2.9276	0.0237
			*Brevundimonas* (Otu092)	3.1321	2.9378	0.0002
Mother’s consumption of probiotics	NO	24	*Acinetobacter* (Otu004)	4.8072	4.4810	0.0137
			*Enhydrobacter* (Otu022)	3.6079	3.4985	0.0365
	YES	7	*Haemophilus* (Otu094)	2.8270	3.1140	0.0040
Baby’s consumption of probiotics	P1	6	*Corynebacterium* (Otu025)	3.2552	3.0337	0.0209
	P2	5	*Lactobacillus* (Otu024)	3.6063	3.2836	0.0366
			*Actinomyces* (Otu075)	3.0170	2.7680	0.0366
			*Microbacterium* (Otu105)	2.5051	2.4253	0.0366
	P^∗^	11	*Lactobacillus*	3.9988	3.9534	0.0110

*P1, *Lactobacillus reuteri* DSM 17938; P2, *Lactobacillus rhamnosus* GG. ^∗^Reads binned according to taxonomic classification.*

**FIGURE 5:**
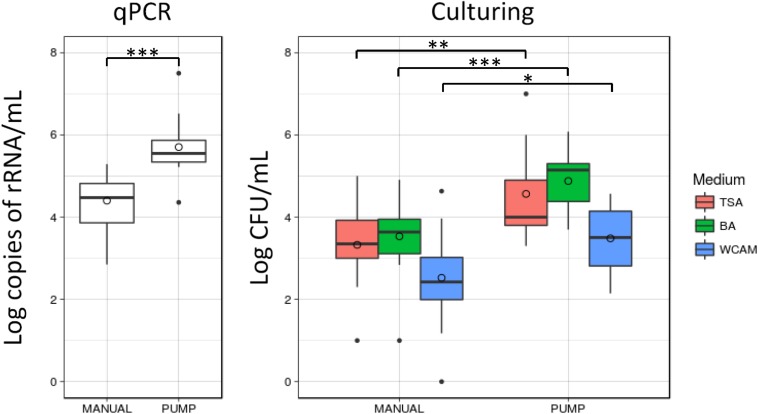
Molecular- and cultivation-based quantification of bacterial load in human milk samples. Samples that mothers collected by manual expression had significantly lower content of bacterial DNA and decreased number of bacteria, grown on BA, TSA, and WCA-M. Statistical significance according to Mann–Whitney rank sum test: ^∗^*p* < 0.05, ^∗∗^*p* < 0.01, ^∗∗∗^*p* < 0.001.

The use of a breast pump to collect the milk sample was significantly associated with specific patterns in HMM, determined with 16S NGS (AMOVA, *p* = 0.049), with several OTUs being significantly increased in the PUMP group and several in the MANUAL group (Table 1). Differentiation in community composition between MANUAL and PUMP groups is presented with NMDS plots (Supplementary Figure S1). Moreover, samples that mothers collected by manual expression had significantly decreased bacterial load; determined by qPCR; and cultivation on BA, TSA, and WCA-M (Figure 5). Culture technique also revealed higher relative abundance of *Staphylococcus* in the MANUAL group, determined by LEfSe (LDA = 5.144, *p* = 0.045) and Mann–Whitney rank sum test (*p* = 0.048; Supplementary Figure S2).

Analysis of the questionnaires showed that 11 mothers gave their infants one of the two probiotic preparations the month before the sampling (Supplementary Table S1). Both of them were lyophilized probiotics in oil suspensions (probiotic drops). One product contained LGG and the other LRD. LEfSe analysis showed that all differentially increased OTUs in relation to the type of supplemented probiotic (Table 1) belong to *Firmicutes* or *Actinobacteria*. In addition, when we binned the reads according to the taxonomic classification at the genus level, we observed a significant increase in the relative abundance of *Lactobacillus* in milk from mothers whose babies were given probiotics (Table 1). Interestingly, neither of seven OTUs classified to genus *Lactobacillus* showed significant increase, when compared between the two groups. With the cultivation approach, we observed prominent differences in prevalence of bacteria grown on ROG agar (Supplementary Table S10). Within the probiotic group, presumable lactobacilli were detected on ROG agar in 63.3% (7/11) of the samples while in the rest of the samples only one was culture positive (5%; Fisher’s exact *p* < 0.001). Moreover, with the strain-specific PCR, we identified cultivable LRD in two samples from the P1 probiotic group and LGG in three samples from the P2 probiotic group and in one sample from the group where the mothers did not declare administration of probiotics to their infants. This result agrees with the oligotype analysis of reads assigned *to Lactobacillus* from 16S NGS – namely, *L. reuteri* or *L. rhamnosus* assigned oligotypes, matched the samples in which live LGG or LRD was detected with strain-specific PCR (Supplementary Table S11). Consumption of probiotics by infants could be associated with specific changes in HMM; however, the number of subjects were low and the effect was assessed on the basis of maternal reports.

## Discussion

The aim of the present study was to characterize HMM with culture-dependent and culture-independent techniques and to evaluate some of the factors that might influence HMM (Garcia-Mantrana et al., 2017). Therefore, we successfully used both, 16S NGS and cultivation/MALDI-TOF MS identification, techniques to address this question. Since accuracy of MALDI-TOF MS determination is limited to the quality of the spectral database (Sauget et al., 2017), we also tested its accuracy with Sanger sequencing of 16S rRNA isolated from selected colonies.

Similar as in our study, several other studies report Firmicutes as predominant phyla for the samples from Western locations, regardless of the technique used (Cabrera-Rubio et al., 2012; Jost et al., 2013a; Ward et al., 2013; Boix-Amoros et al., 2016; Kumar et al., 2016; Urbaniak et al., 2016; Murphy et al., 2017; Williams et al., 2017a). Additionally, studies from India, China, and Taiwan report the predominance of *Proteobacteria* (Li et al., 2017; Patel et al., 2017; Vaidya et al., 2017; Xi et al., 2017) which suggest a geographical area-dependent microbiota (Kumar et al., 2016; Li et al., 2017). The notable difference in results from the culture-dependent and -independent techniques was in the abundance of *Proteobacteria* and *Actinobacteria* which is in agreement with results from Jost et al. (2013a). Explanations for these differences lie either in the culture bias due to favored growth of non-fastidious microorganisms or in the molecular detection of non-cultivable bacterial cells (Jost et al., 2013a).

Our results are in concordance with the previous NGS studies, which report *Staphylococcus* and/or *Streptococcus* as predominant genera (Hunt et al., 2011; Jimenez et al., 2015; Boix-Amoros et al., 2016; Cabrera-Rubio et al., 2016; Kumar et al., 2016; Sakwinska et al., 2016; Urbaniak et al., 2016; Li et al., 2017; Williams et al., 2017a; Chen et al., 2018; Meehan et al., 2018; Simpson et al., 2018). Others also reported *Pseudomonas* (Jost et al., 2013a, b; Ward et al., 2013; Murphy et al., 2017), *Acinetobacter* (Patel et al., 2017), *Leuconostoc* (Cabrera-Rubio et al., 2012), or *Enterobacteriaceae* family (Vaidya et al., 2017) as predominant. However, only *Staphylococcus* and *Streptococcus* were commonly listed as predominant genera, which suggest that only these two are a part of the “core HMM” (Fitzstevens et al., 2017; Simpson et al., 2018). It is worth noting that although many studies report *Pseudomonas* as highly abundant genus in milk samples the prevalence of *Pseudomonas* and several other OTUs in our study is highly questionable. Similar observations were reported from Jost et al. (2013a) where high abundance of *Pseudomonas* and *Ralstonia*, detected with molecular techniques, also failed to be confirmed with culture approach.

Previous culture-dependent studies largely confirm the predominance of *Staphylococcus*, *Streptococcus*, and *Propionibacterium* spp. (Heikkila and Saris, 2003; Jimenez et al., 2008; Solis et al., 2010; Jost et al., 2013a). It is interesting to note that in several samples, the 16S NGS approach did not confirm the presence of cultivable genera like *Propionibacterium*, *Bacillus*, *Micrococcus*, *Bifidobacterium*, *Ochrobactrum*, *Kocuria*, *Moraxella*, and *Klebsiella*. Low concentration of a certain strain and their selective growth on the specific agars on the one hand and bias introduced during library preparation and sequencing on the other hand may be the reason for these discrepancies.

*Staphylococcus* was traditionally considered as a major mastitis-causing pathogen (Fernandez et al., 2014) and the abundance of *Staphylococcus* was considerably higher in subacute and acute mastitis cases compared to healthy controls. Changes in the HMM were also accompanied by the loss of bacterial diversity (Jimenez et al., 2015; Patel et al., 2017). The observed negative correlation between Shannon diversity index and the abundance of *Staphylococcus* (Figure 4) seems to be in agreement with the dysbiosis model of mastitis, where overgrowth of one species lead to decreased bacterial diversity, accompanied by dramatic enrichment of aerotolerant bacteria and depletion of obligate anaerobes (Angelopoulou et al., 2018). The inclusion criteria in our study were only self-reported healthy mothers – however, we cannot exclude possible subacute mastitis cases or tendency to mastitis in individual mothers.

Milk is also one of the most important foods and MALDI-TOF MS was successfully applied for determination of mastitis-causing pathogens, various spoilage bacteria and lactic acid bacteria used as probiotics (Hess et al., 2016). The rationale for usage of MALDI-TOF MS for HMM characterization lies in the fact that the MALDI-TOF MS spectral database contains a variety of spectra of bacteria that are usually isolated from milk samples. Indeed, MALDI-TOF MS proved to be a suitable tool for determination of HMM. The only limitation was the background spectra of WCA-M agar, which led to false positive detection of *Mycoplasma.* The effect was more pronounced when we tried to determine low-biomass colonies grown on WCA-M. Balazova et al. (2014) reported that the residues of the cultivation medium could interfere with the MALDI-TOF MS analysis.

Several recent studies have pointed out maternal and external factors that could potentially influence HMM (Gomez-Gallego et al., 2016; Garcia-Mantrana et al., 2017). In agreement with recent studies (Urbaniak et al., 2016; Li et al., 2017;  Williams et al., 2017a), our study showed no significant correlations between HMM and baby’s gender or mother’s age. The correlation between maternal BMI and HMM was previously reported (Cabrera-Rubio et al., 2012; Kumar et al., 2016; Williams et al., 2017a). However, similar to Li et al. (2017), our study did not find significant effects of BMI on HMM. A possible explanation for these differences might be the fact that we did not analyze obese mothers with higher BMI, where the effects on HMM could be more pronounced – namely, the majority (95%) of mothers in our study had BMI between 20.4 and 29.3.

In present observational study, a small proportion of mothers (7/31) reported consumption of different types of probiotics (mostly probiotic yogurts) during the breastfeeding period. Although the HMM of mothers who claimed to consume probiotics had a significantly increased relative abundance of several OTUs, the observed differences are most probably due to unequal distribution between the two groups, and due to overlapping with other effects. For instance, mothers who did not consume probiotics and had high relative abundance of *Acinetobacter* (Otu004) also used breast pumps. Since breast pumps might be the additional source of *Acinetobacter* (Sakwinska et al., 2016), higher relative abundance of *Acinetobacter* (Otu004) in the non-probiotic group could be due to the use of breast pumps.

The milk collection technique had a significant influence on HMM in several ways. In agreement with Marin et al. (2009), the PUMP group had higher bacterial counts in different culture media and in our case higher content of bacterial DNA. Most probably, cultivable staphylococci found in samples from the MANUAL group originated from mothers’ hands and skin, where they are part of the commensal microbiota (Byrd et al., 2018). In regard to 16S NGS analysis, the common denominator of OTUs that were significantly increased in the MANUAL group is that they can all be considered as contaminant OTUs, since they appeared also in the experimental or library preparation controls (Supplementary Table S2). This result is most probably the consequence of lower content of bacterial DNA in the MANUAL group and subsequent increase of contaminant OTUs. The contaminant OTUs are usually more prominent in samples with lower microbial load (Glassing et al., 2016). The OTUs increased in the PUMP group can be classified as environmental bacteria, originating from the environment or rinsing water. It is reasonable to assume that contaminating bacterial DNA, arising from the environment, rinsing water, or poor hygienic practices, may persist in milk pumps and/or their accessories, since domestic cleaning protocols such as soap and boiling do not ensure degradation of DNA (Escuder-Vieco et al., 2018). Moreover, mothers often do not follow the guidance for milk collection, leading to inappropriate cleaning of the breast pump (Boo et al., 2001; Carre et al., 2018). Recent study from Moossavi et al. (2019) confirmed that “mode of breastfeeding” as a major impact factor that influence on HMM. Namely, pumped breastmilk was associated with several HMM parameters, such as higher abundance of potential pathogens and lower abundance of bifidobacteria. These data further support the so called “retrograde inoculation hypothesis,” by which the infant oral cavity and oral microbiota influences on the HMM (Moossavi et al., 2019).

Recent findings from Biagi et al. (2018) showed that HMM composition was more diverse and dominated by typical oral bacteria such as *Streptococcus* and *Rothia*, after the infant’s latching to the mother’s breast. Theoretically, specific probiotics present in the infant’s oral cavity and saliva could also be transferred to the mother’s milk with retrograde flow during suckling. In the light of retrograde inoculation hypothesis indeed, our results from both, culture-dependent and culture-independent techniques indicate that the administration of probiotics to infants, might influenced HMM, although the number of samples in each subgroup was small; therefore, further investigation is required for more reliable conclusions. We hypothesize that the administration of probiotics in oil suspension could influence HMM via retrograde flow during suckling. We can assume that oil suspension also facilitates the probiotic to persist a longer time in the oral cavity of the baby, leading to inoculation of the mammary gland. Nonetheless, further, more targeted investigations are needed to confirm any of these speculations.

Several limitations of this study should be taken into consideration. Most importantly, this was an observational study and mothers were asked to sample their milk at home. Thus, contamination with the probiotics and other surrounding bacteria due to improper cleaning of the breast or breast pump cannot be excluded. Another limitation regarding sample collection protocol was freezing the samples before the analysis, which might introduce potential bias in results from cultivation/MALDI-TOF MS, although short-term freezing should not substantially impact on HMM (Marin et al., 2009). It should be noted that beside inherent limitations of culture-dependent methods, major limitation of cultivation/MALDI-TOF MS was the number of analyzed colonies and limited number of different growth media and growth conditions used. With increasing the up-mentioned parameters, we could cover greater diversity of cultivable part of HMM. Since contaminant DNA in 16S NGS seems to be unavoidable problem, especially in low biomass samples (Tobin et al., 2018), we decided to present all bacterial groups which were present in our samples – however, we urge the reader to consider the possibility that the highlighted (^∗^) bacterial groups are contaminants. Another important limitation of our study was the collection of the sample metadata by short questionnaire in retrospective manner, which could lead to false negative or positive reporting. For example, false negative reporting on consumption of probiotics in the non-probiotic group of mothers could explain the presence of viable LGG strain in the non-probiotic group (sample s09). As discussed above, relatively small number of participating mothers could also lead to less powerful statistics and to less solid conclusions.

## Conclusion

As shown in previous studies, culture techniques offer important supplementation to modern culture independent NGS data. Here we showed that cultivation/MALDI-TOF MS was a suitable tool for determination of HMM. *Staphylococcus* and *Streptococcus* were the predominant genera in HMM and the abundance of *Staphylococcus* was associated with decreased microbial diversity. The use of a breast pump is an important factor regarding milk collection technique. Our study suggests that administration of probiotics to the suckling infant might influence HMM by increased abundance of lactobacilli and the presence of viable probiotic bacteria in human milk. However, since our study was observational with relatively small sample size, more targeted studies are needed to study possible transfer of probiotics to the mammary gland via an external route and the physiological relevance of these events.

## Data Availability Statement

The datasets (generated) for this study can be found in the Metagenomics RAST, project access number mgp88161 (https://www.mg-rast.org/mgmain.html?mgpage=project&project=mgp88161).

## Ethics Statement

The studies involving human participants were reviewed and approved by the National Medical Ethics Committee of the Republic of Slovenia (approval number: 0120-328/2017/3). The patients/participants provided their written informed consent to participate in this study.

## Author Contributions

PT recruited the subjects, collected the samples, performed the qPCR analysis, and wrote the manuscript. PT and PL designed the study. PL prepared the samples for the microbial analysis, isolated the microbial DNA, and performed the culture-dependent analysis. AM performed the 16S NGS and the statistical analysis, and analyzed the 16S NGS data. MR helped with statistical analysis and manuscript review. MG and TP performed the MALDI-TOF MS analysis of the samples. PT and BM provided the financial support. BM helped with the study design and manuscript writing. All authors read and approved the final version of the manuscript.

## Conflict of Interest

The authors declare that the research was conducted in the absence of any commercial or financial relationships that could be construed as a potential conflict of interest.
