# Corrigendum: Evaluation of Human Milk Microbiota by 16S rRNA Gene Next-Generation Sequencing (NGS) and Cultivation/MALDI-TOF Mass Spectrometry Identification

**DOI:** 10.3389/fmicb.2020.00031

**Published:** 2020-01-29

**Authors:** Primož Treven, Aleksander Mahnič, Maja Rupnik, Majda Golob, Tina Pirš, Bojana Bogovič Matijašić, Petra Mohar Lorbeg

**Affiliations:** ^1^Department of Animal Science, Biotechnical Faculty, Institute of Dairy Science and Probiotics, University of Ljubljana, Ljubljana, Slovenia; ^2^National Laboratory of Health, Environment and Food, Maribor, Slovenia; ^3^Faculty of Medicine, University of Maribor, Maribor, Slovenia; ^4^Faculty of Veterinary Medicine, Institute of Microbiology and Parasitology, University of Ljubljana, Ljubljana, Slovenia

**Keywords:** milk microbiota, microbiota assembly, breastfeeding, breast pump, probiotic drops

In the original article, there was an error. The line in the Abstract section: “The use of a breast pump was significantly associated with composition of HMM, lower microbial load, and higher abundance of cultivable staphylococci,” was not correct.

A correction has been made to the Abstract: “The use of a breast pump was significantly associated with composition of HMM, higher microbial load, and lower abundance of cultivable staphylococci.”

The authors apologize for this error and state that this does not change the scientific conclusions of the article in any way. The original article has been updated.

