# Supplementary material for: Evaluation of Human Milk Microbiota by 16S rRNA Gene Next-Generation Sequencing (NGS) and Cultivation/MALDI-TOF Mass Spectrometry Identification
Source: Front Microbiol. 2019 Nov 15;10:2612. doi: 10.3389/fmicb.2019.02612 (PMC6872673; doi:10.3389/fmicb.2019.02612)
Supplement: Supplementary file 2 [file Data_Sheet_2.docx]

Supplementary Material

# Supplementary file 2:

Fig S1: NMDS presentation of Bray–Curtis dissimilarity shows differences in community composition between the MANUAL and the PUMP group determined by 16S NGS.

Fig S2: Differences in *Staphylococcus* relative abundance determined by cultivation and MALDI-TOF MS identification in relation to the use of a breast pump to collect the milk sample.


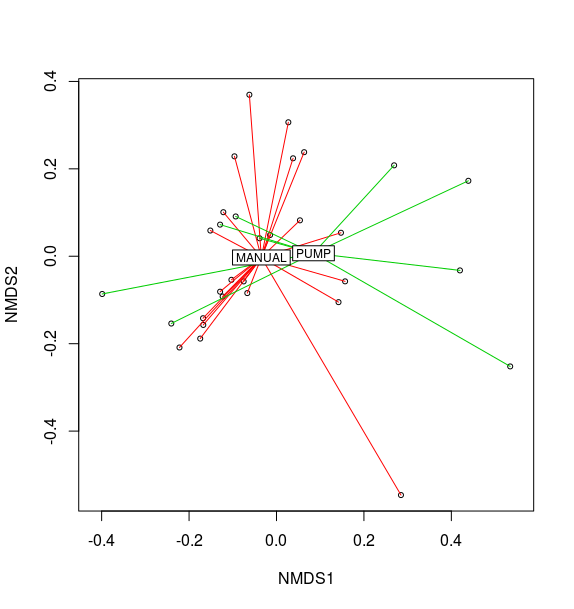


AMOVA, p=0.049

**Fig. S1: NMDS plots using Bray–Curtis distance matrices show differences in community composition between the MANUAL and the PUMP group determined by 16S NGS.**

*

**Fig. S2: Differences in Staphylococcus relative abundance determined by cultivation and MALDI-TOF MS identification in relation to the use of a breast pump to collect the milk sample.***Statistical significance according to Mann-Whitney Rank Sum Test: p<0.05.
